# Hollow-Structure Pt-Ni Nanoparticle Electrocatalysts for Oxygen Reduction Reaction

**DOI:** 10.3390/molecules27082524

**Published:** 2022-04-14

**Authors:** Quan Wang, Baosen Mi, Jun Zhou, Ziwei Qin, Zhuo Chen, Hongbin Wang

**Affiliations:** 1 School of Materials Science and Engineering, Shanghai University, Shanghai 200444, China; wangquan1619@163.com (Q.W.); baosenmi@shu.edu.cn (B.M.); janszhou@126.com (J.Z.); zwqin@t.shu.edu.cn (Z.Q.); chenzhuo@shu.edu.cn (Z.C.); 2 H2E Technology Zhejiang Co. Ltd., Jinhua 321000, China; 3 Shanghai Engineering Research Center for Metal Parts Green Remanufacture, Shanghai 200444, China

**Keywords:** electrocatalyst, oxygen reduction reaction, PEMFC, hollow structure, carbon-free

## Abstract

An electrocatalyst with high oxygen reduction reaction (ORR) activity and high stability during start–stop operation is necessary. In this paper, hollow-structure Pt-Ni electrocatalysts are investigated as ORR catalysts. After synthesis via sacrificial SiO_2_ template method, the electrocatalyst exhibits much higher specific activity (1.88 mA/cm^2^) than a commercial Pt/C catalyst. The mass activity (0.49 A/mg) is 7 times higher than the commercial Pt/C catalyst. The kinetics of the ORR is evaluated using Tafel and K-L plots. It also exhibits a higher durability than commercial Pt/C catalyst during accelerated durability test (ADT). Moreover, the electrocatalyst shows good resistance against accelerated durability test for start–stop, the specific activity and mass activity drops 34.6% and 40.8%, respectively, far better than the commercial catalyst.

## 1. Introduction

Hydrogen has attracted much attention as a kind of clean energy. Moreover, it is expected to become the next generation of new energy [1]. Proton exchange membrane fuel cell (PEMFC) is a device which converts hydrogen into electricity. However, the sluggish kinetics of the oxygen reduction reaction (ORR) process at the cathode and the high price of noble metal catalysts have limited the commercialization of PEMFC [2,3]. In this regard, many researchers have prepared electrocatalysts with different structures to improve the ORR activity in the cathode, such as porous structure, hollow structure, dodecahedral nanocrystal, nanowire, alloy nanoframe and nanoplate [4,5,6,7,8,9]. To a certain extent, these structures of catalysts have improved ORR activity by modifying the geometric and electronic structures of Pt, such as d-band center, surface strain effect and coordination numbers composited with transition metals such as Fe, Co, Ni.

Even though the catalysts above have shown good performance, most of them were supported by carbon. It is easy to cause carbon corrosion during start–stop operations in PEMFCs [10]. When this happens, the electrocatalysts agglomerate due to the loss of high-surface-area carbon, eventually leading to degradation failure of the catalyst. If the carbon corrosion issue is solved, the durability of Pt-based catalyst can be greatly improved while maintaining ORR activity. Moreover, the control system of PEMFC vehicles will be simplified if there is no carbon corrosion occurs [11]. Therefore, the prevention of carbon corrosion is an important research direction nowadays. Recently, the carbon-free catalyst has given a new possibility to solve this problem [12,13,14]. For example, the Pt-based carbon-free catalyst Pt/Ti_3_O_5_-Mo shows a good performance with specific activity (SA) about 1.1 mA/cm^2^ at 0.9 V vs. RHE, and there was only 11.2% electrochemical surface area (ECSA) loss after 5000 potential cycles [12]. However, PtCo core-shell nanoparticles based on carbon reached 30% ECSA loss after 5000 potential cycles, with a similar SA to the carbon-free catalyst [15]. This proved that the durability can be improved effectively by using a carbon-free catalyst.

Therefore, hollow-structure Pt-Ni nanoparticle electrocatalysts were synthesized by sacrificial template method as the carbon-free catalyst on the cathode of PEMFCs. The high surface area of the hollow structure can guarantee the ORR activity of the catalyst. In addition, the structure can effectively avoid the loss of performance caused by carbon corrosion, since no carbon is needed. Then, its structure is evaluated and its electrochemical activity toward ORR is studied in detail.

## 2. Results and Discussion

### 2.1. Structure and Characterization of Pt-Ni Electrocatalyst

**Figure 1:**
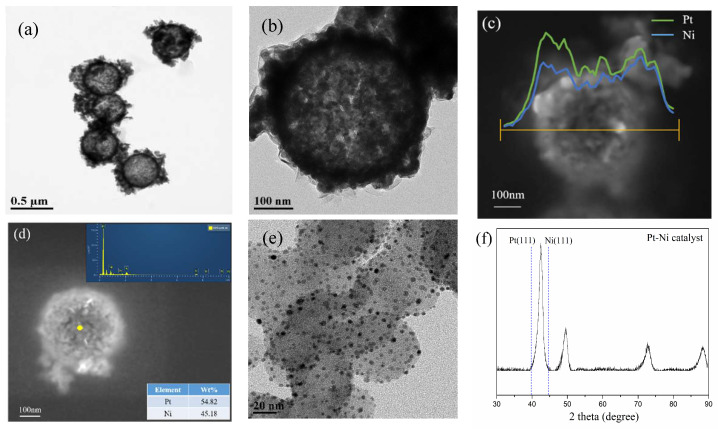
(**a**,**b**) TEM image of the Pt-Ni electrocatalyst, (**c**) SEM image of the Pt-Ni electrocatalyst and the composition of the electrocatalyst determine by EDS, (**d**) EDS result of the Pt-Ni electrocatalyst, (**e**) TEM image of 20% Pt/C catalyst, (**f**) XRD pattern of hollow-structure Pt-Ni electrocatalyst.

Figure 1a,b shows the TEM image of the Pt-Ni electrocatalyst. Most of Pt-Ni nanoparticles are spherical with a diameter of about 370 nm. It is calculated that the thickness of the catalytic layer is about 35 nm because the average diameter of the silica sphere template is 300 nm. It can also be proven in Figure 1b by the TEM image. In addition, Figure 1c shows that nanoparticles are hollow structures composed of Pt and Ni. The Pt-Ni weight ratio is about 1.2:1 and the Pt and Ni are uniformly distributed in the nanoparticle. In addition, the EDS result is shown in Figure 1d. The results show that the content of Pt in the nanoparticle is 54.82 wt%, and the rest is Ni. For comparison, the TEM image of 20% Pt/C is shown in Figure 1e.

The XRD pattern of the Pt-Ni electrocatalyst is shown in Figure 1f. The pattern shows that Pt-Ni hollow-structure electrocatalyst exhibits a face-centered cubic (fcc) phase, without the formation of ordered phase. The 2 theta degree of Pt-Ni (111) peak is 42.5°, which is between Pt (111) and Ni (111), and the lattice constant of Pt-Ni catalyst is 0.367 nm while the lattice constants of metal Pt and Ni are 0.392 nm and 0.352 nm, respectively. When Ni is added, lattice contraction occurs due to Ni atoms entering Pt lattice, which shifts the peak toward a higher angle. The rules of the lattice constant and peak obtained by XRD are consistent with previous work on core-shell PtNi catalyst [16]. The crystallite size of the Pt-Ni electrocatalyst, calculated using Scherrer formula, is approximately 6.5 nm.

**Figure 2:**
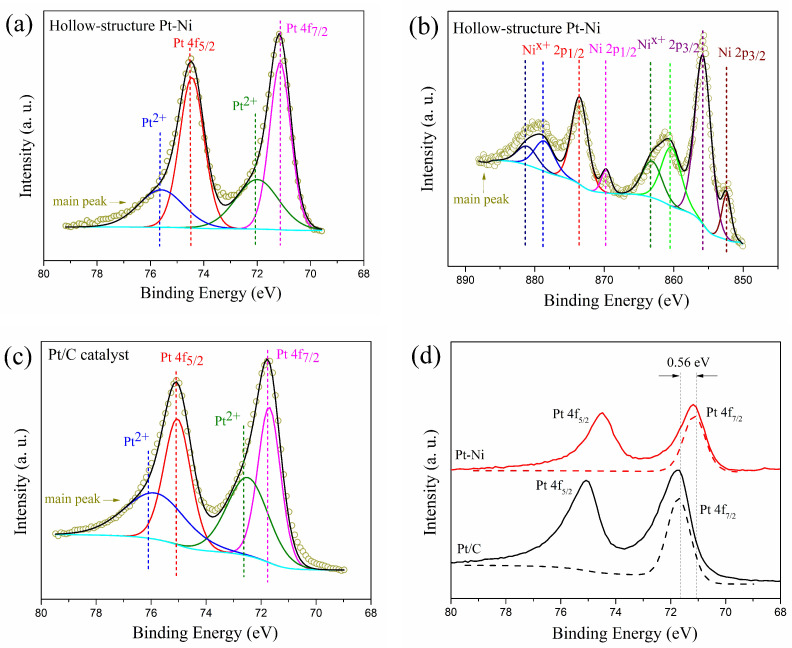
(**a**) surface Pt 4f XPS spectra pattern of hollow-structure Pt-Ni electrocatalyst, (**b**) surface Ni 2p XPS spectra pattern of hollow-structure Pt-Ni, (**c**) surface Pt 4f XPS spectra pattern of 20% Pt/C catalyst, (**d**) comparison of Pt 4f_7/2_ peaks of Pt-Ni and 20% Pt/C catalysts.

Figure 2 shows the surface characteristics of the Pt-Ni electrocatalyst. The Pt 4f XPS spectra and Ni 2p XPS spectra of Pt-Ni electrocatalyst are shown in Figure 2a,b. The XPS spectra patterns indicate that the surface Pt is mainly in the metallic state and that the surface Ni is mainly in the oxidized state. These results are consistent with Pt-Ni catalysts studies [17,18]. It has been shown in previous studies that nickel and oxygen have a higher affinity [19,20]. This indicates that the oxidation of Ni takes precedence over Pt during Pt-Ni electrocatalyst preparation. As a result, more Pt will exist in metallic state to ensure catalytic performance of the electrocatalyst. The Pt 4f XPS spectra of Pt/C catalyst is shown in Figure 2c. The surface Pt of Pt/C is mainly in the metallic state which has the similar tendency of Pt-Ni catalyst, while the peaks of Pt 4f_7/2_ are quite different. In Figure 2d, the binding energy of metallic state of two catalysts for Pt 4f_7/2_ peaks are 71.14 eV for Pt-Ni hollow-structure catalyst and 71.7 eV for Pt/C catalyst. The results show that the binding energy of metallic state of Pt-Ni hollow-structure catalyst Pt 4f_7/2_ peak is negatively shifted. It indicates that the addition of Ni causes electron orbital overlap and changes the electron density on the surface of Pt [21,22]. The binding energy is 0.56 eV negatively shifted indicates that increases the electron density of Pt atom on the surface and reduces the binding energy of O adsorption [22,23,24,25]. This promotes the oxygen reduction reaction performance of catalyst.

### 2.2. Electrochemical Characterization Analysis

**Figure 3:**
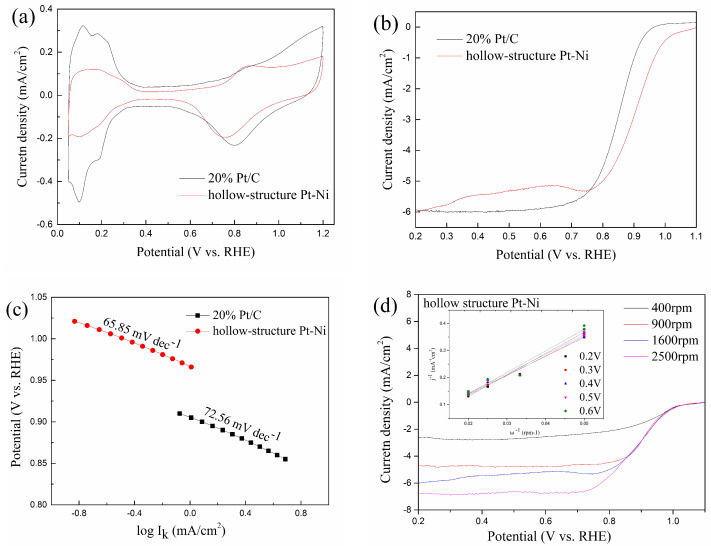
(**a**) cyclic voltammetry curves of hollow-structure Pt-Ni electrocatalyst and 20% Pt/C catalyst, (**b**) LSV polarization curves of hollow-structure Pt-Ni electrocatalyst and 20% Pt/C catalyst, (**c**) Tafel plot of hollow-structure Pt-Ni electrocatalyst and 20% Pt/C catalyst, (**d**) LSV polarization curves of hollow-structure Pt-Ni electrocatalyst with various rotation speeds, the corresponding Koutechky–Levich plots at various voltages are shown in the inset.

Cyclic voltammograms of 20% Pt/C and hollow-structure Pt-Ni are shown in Figure 3a. As it shown, the hydrogen adsorption region of hollow-structure Pt-Ni is obviously smaller than Pt/C. The electrochemical active surface area (ECSA) of 20% Pt/C is calculated to be 58.8 m^2^/g, within the range of the values for commercial Pt/C catalysts [26,27]. ECSA of hollow-structure Pt-Ni is 25.9 m^2^/g, which is only 44.1% of commercial Pt/C. This is because commercial Pt/C has highly dispersed 3–5 nm Pt nanoparticles on the carbon support [28], it is also shown in Figure 1e. While the hollow-structure Pt-Ni is about 370 nm and the Pt nanoparticles is 6.5 nm calculated by Scherrer formula, which is larger than commercial Pt/C. 

To evaluate the ORR activities, the LSV curves are obtained by rotating disk electrode in 0.1 M HClO4, as shown in Figure 3b. The values of current density at 0.9 V vs. RHE are 1.02 mA/cm^2^ and 2.89 mA/cm^2^ for Pt/C and hollow-structure Pt-Ni, respectively. The kinetic current ($I_{k}$) of Pt/C and hollow-structure Pt-Ni are calculated by Formula (2). In order to evaluate the performance of electrocatalyst, and to compare the mass activity (MA) and specific activity (SA) with different catalysts, the *I_k_* is normalized to both the loading amount of metal and ECSA, respectively. The specific activity of Pt/C is 0.1 mA/cm^2^, which is a little lower than the reported values for commercial Pt/C catalysts [29,30]. The experiment has been repeated several times, but with similar results. Therefore, it is most probable that the commercial catalysts purchased have low performance. The hollow-structure Pt-Ni exhibits a higher specific activity of 1.88 mA/cm^2^ than that of the Pt/C. Furthermore, the hollow-structure Pt-Ni shows a mass activity of 0.49 A/mg, which is also greater than that of the Pt/C catalyst (0.07 A/mg). The results show that the ORR activity of hollow-structure Pt-Ni is better than that of Pt/C catalyst. The most possible reason for the improvement is that the hollow structure helps expand the surface of the catalyst and promotes the ORR process.

Furthermore, the Tafel plot of the 20% Pt/C and hollow-structure Pt-Ni are shown in Figure 3c. As the result shown, the Tafel slope of the hollow-structure Pt-Ni is 65.85 mV/dec, which is lower than that of the 20% Pt/C (72.56 mV/dec, similar with the results in References [31,32]). Generally, the Tafel slope can be lowered due to the accelerated ORR kinetics [33]. Therefore, referring to the previous LSV experiment, it is further proved that the hollow-structure Pt-Ni electrocatalyst has a better ORR activity than 20% Pt/C.

As is shown, the O_2_ is reduced to H_2_O_2_ via two-electron process and H_2_O via four-electron process [34]. H_2_O_2_ formed on the electrodes can diffuse into the membrane, leading to the gradual loss of membrane materials, so the four-electron ORR pathway is desired [35]. Therefore, to evaluate the number of electrons transfer, a further analysis of kinetics of the hollow-structure Pt-Ni electrocatalyst for ORR activity is obtained by Koutecky-Levich (K-L) theory. The LSV at various rotation speeds and the K-L plots are shown in Figure 3d. The formula and the constants for calculation are detailed in Section 3.4. The slopes of K-L plots for the hollow-structure Pt-Ni electrocatalyst are approximately in the voltage range from 0.2 V to 0.6 V vs. RHE. It reveals that the number of electrons transferred for ORR at various voltages are similar, which are 3.37, 3.67, 3.8, 3.69, and 3.34, respectively. It suggested that the electron transfer of ORR process is controlled by a combination of two-electron and four-electron transfer pathway.

### 2.3. Electrocatalysts Durability Analysis by Electrochemical Method

**Figure 4:**
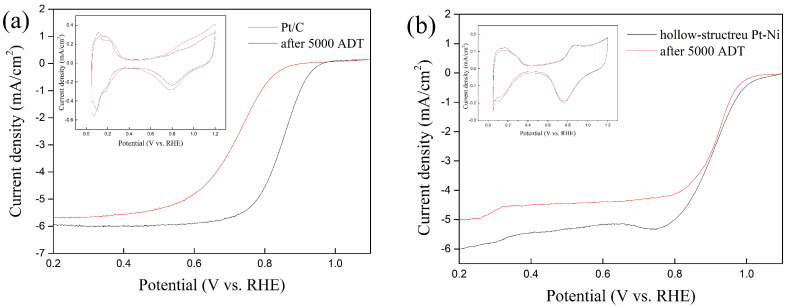
(**a**) CV and LSV polarization curves of 20% Pt/C catalyst before and after 5000 cycles ADT, (**b**) CV and LSV polarization curves of hollow-structure Pt-Ni electrocatalyst before and after 5000 cycles ADT.

In order to evaluate the durability of the hollow-structure Pt-Ni electrocatalyst, an accelerated durability test (ADT) is employed. The results of before and after 5000 cycles ADT for both Pt/C and hollow-structure Pt-Ni are shown in Figure 4a,b. After 5000 cycles ADT, the ECSA of hollow-structure Pt-Ni is similar to that before ADT. While the Pt/C (57.1 m^2^/g) shows a loss of 2.8% during the same process. For SA and MA, the activity of hollow-structure Pt-Ni drops by 13.8% and 28.5% respectively, but the ORR activities of commercial catalyst can hardly be measured at 0.9 V vs. RHE. Thus, the hollow-structure Pt-Ni shows more stability than the Pt/C catalyst. The reason is that the Pt/C with ultrafine nanoparticles will occur movement, aggregation and Ostwald ripening during the ADT process [36,37]. When the Pt particles in Pt/C grow up, the ORR activity of Pt/C decreases obviously. On the contrary, the hollow structure shows more stability against the ADT to avoid movement, aggregation and Ostwald ripening, so that the ORR activity still remains at a good level after ADT.

**Figure 5:**
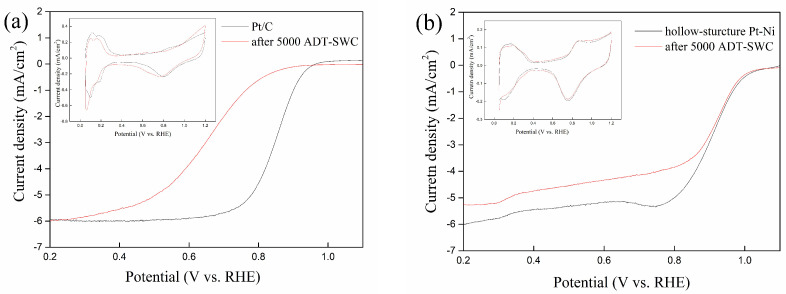
(**a**) CV and LSV polarization curves of 20% Pt/C catalyst before and after 5000 cycles of ADT-SWC, (**b**) CV and LSV polarization curves of hollow-structure Pt-Ni electrocatalyst before and after 5000 cycles of ADT-SWC.

In addition, hollow-structure Pt-Ni electorcatalysts are designed to resist the carbon corrosion in the start-stop operation in PEMFC, so the start–stop durability test by square wave potential cycles (ADT-SWC) is employed. The results of before and after 5000 of ADT-SWC for both Pt/C and hollow-structure Pt-Ni are shown in Figure 5a,b. After 5000 cycles of ADT-SWC, the ECSA of hollow-structure Pt-Ni is 23.4 m^2^/g, a drop of about 9.6%. However, the ECSA of Pt/C is dropped to 38.3 m^2^/g, even lower than the Pt/C after 5000 cycles of ADT. The possible reason for this is that carbon corrosion during the start-stop process will promote particle growth and aggregation [38]. Furthermore, the ORR activity is seriously affected by particle size and distribution, so the Pt/C exhibits severely worse activity after 5000 cycles of ADT-SWC. The SA and MA of hollow-structure Pt-Ni after 5000 cycles of ADT-SWC are 1.23 mA/cm^2^ and 0.29 A/mg, respectively; these are only slightly different from the initial SA and MA value of the hollow-structure Pt-Ni electrocatalyst, but still better than those of the commercial catalysts. It shows the hollow-structure Pt-Ni electrocatalyst owns better stability against start–stop operation. All the data about the LSV of Pt/C and hollow-structure Pt-Ni electrocatalyst before and after ADT and ADT-SWC are detailed in Table 1. To evaluate the activities of the as-prepared hollow-structure Pt-Ni electrocatalyst, a comparison is made with some previous works (the MA of these works are from 0.19 to 0.85 A/mg, and the SA are from 0.56 to 2.27 mA/cm^2^) [4,16,39,40,41,42]. Compared to these works, the ORR activities of the as-prepared electrocatalyst is at a good level.

## 3. Materials and Methods

### 3.1. Materials

Platinum (II) acetylacetonate (Pt(acac)_2_), Nickel (II) acetylacetonate (Ni(acac)_2_), poly dimethyl diallyl ammonium chloride (PDDA), tetraethylene glycol and isopropanol alcohol were purchased from Rhawn. (Shanghai, China). Silica spheres were purchased from Shuangying Alloy Material (Shanghai, China). Ethanol and sodium hydroxide (NaOH) were purchased from Sinopharm Chemical Reagent Co., Ltd. (Shanghai, China). Deionized water was used in all cases. Those chemicals were of analytical grade and used without further purification.

For the electrochemical evaluation, analytical grade perchloric acid (HClO_4_) was purchased from Sinopharm Chemical Reagent Co., Ltd. (Shanghai, China). 5% Nafion solution was obtained from Dupont (Wilmington, DE, USA). In addition, 20% Pt/C was used as standard electrocatalysts for the comparison in this paper and purchased from Jiping New Energy Technology Co., Ltd. (Shanghai, China).

### 3.2. Synthesis of Hollow-Structure Pt-Ni Nanoparticles

The hollow-structure electrocatalysts were synthesized via sacrificial template method [43,44]. The details of the synthesis are as follows: 32 mg of silica spheres with diameters of about 300 nm were dispersed in 5 mL water and 10 mL PDDA solution. The mixture was ultrasonic dispersed at room temperature for 10 min. PDDA-modified silica spheres were centrifuged three times with deionized water. Then the modified silica spheres were dispersed in 80 mL tetraethylene glycol. After that, 125.8 mg Pt(acac)_2_ (0.32 mmol) and 82 mg Ni(acac)_2_ (0.32 mmol) were added into tetraethylene glycol, transferred the mixture to a 100 mL three-necked flask when stirred uniformly. The flask was heated with a heating mantle. The mixture was heated from room temperature to 513 K at a heating rate of 10 K per minute and then kept 513 K for 2 h by refluxing with constant stirring. The mixture was cooled to room temperature after the reaction. Then, the compound attached to the silica sphere was collected by centrifugation and washed by ethanol three times, before being dried under room temperature as powder. 

The powder was sealed in a glass tube filled with argon then heated to 623 K in a resistance furnace for 4 h, and subsequently cooled to room temperature. Whereafter, the powder was stirred in 3 mol/L NaOH aqueous solution for 1 h at 353 K to dissolve the silica sphere then washed several times by deionized water. Lastly, hollow-structure Pt-Ni electrocatalysts were centrifuged and dried at room temperature. 

### 3.3. Electrocatalyst Physical Characterization

The size and morphology of the electrocatalyst was obtained by transmission electron microscopy (TEM, JEM-2100F), in addition, the scanning electron microscopy (SEM, Sigma 300, ZEISS) and energy dispersive spectroscopy (EDS, Oxford, UK) were used for electrocatalyst composition analysis. The structural characterization was adopted X-ray diffraction (XRD, D/MAX2200V) with Cu Kα radiation. The electrocatalyst surface characterization was investigated by X-ray photoelectron spectroscopy (XPS, Escalab 250Xi).

### 3.4. Electrochemical Characterization

The electrochemical characterization of the electrocatalyst was tested by electrochemical work station (Gamry Instruments Reference 600), to evaluate the ORR activity of electrocatalyst. A typical three-electrode cell was used. A saturated calomel electrode (SCE) and Pt wire were utilized as reference and counter electrodes, respectively. The ORR activity was performed on a rotating disk electrode (RDE) as the working electrode. The working electrode was prepared as follows. For the hollow-structure Pt-Ni electrocatalyst, 1 mg catalyst was dispersed in 1 mL 25% aqueous isopropyl alcohol solution and 10 μL 5% Nafion solution as the ink, before 7.2 μL ink was dropped on a mirror-polished glassy carbon (GC) electrode with an area of 0.196 cm^2^ and dried at room temperature. The working electrode preparation process for standard 20% Pt/C catalyst was roughly the same. In particular, the formula for the ink was 2 mg 20% Pt/C, dispersed in 1 mL 20% aqueous isopropyl alcohol solution and 10 μL 5% Nafion solution, then 9.8 μL ink was dropped on GC. Since HClO_4_ electrolyte solution showed more similarity to the actual environment of PEMFCs [15,17,45], all the electrochemical tests were carried out in 0.1 M HClO_4_ electrolyte solution.

For the electrochemical process, the electrochemical active surface area (ECSA) was associated with the active sites of the electrocatalyst surface [46]. It was measured by cyclic voltammetry (CV) at 0.1 M N_2_-saturated HClO_4_ electrolyte solution. The potential was swept between 0.05 V and 1.2 V vs. RHE at a sweep rate of 20 mV/s. Before CV measurement, 50 cycles of activation were performed at sweep rate of 100 mV/s. Then, ECSA was calculated by the underpotential deposited hydrogen adsorption ($Q_{h}$) charge collected in the hydrogen adsorption region from 0.05 V to 0.4 V vs. RHE, which was followed Formula (1) [26].

(1) $$\mathrm{ECSA}=\frac{Q_{h}}{m\times q_{h}}$$

where, m is the metal loading, and $q_{h}$ is the charge required for monolayer adsorption of hydrogen on Pt surfaces as 210 μC/cm^2^.

ORR activity including mass activity (MA) and specific activity (SA) were measured by liner sweep voltammetry (LSV) at 0.1 M O_2_-saturated HClO_4_ electrolyte solution. The potential was swept from 0.05 V to 1.1 V vs. RHE at a sweep rate of 10 mV/s and a rotation speed of 1600 rpm. Then the MA and SA of the electrocatalyst was calculated at 0.9 V vs. RHE via the mass transport corrected kinetic current ($I_{k}$) in Formula (2) [27].

(2) $$I_{k}=\frac{I_{lim}\times I}{I_{lim}-I}$$

$I_{lim}$ is the limiting current measured at 0.4 V vs. RHE and I is the current measure at 0.9 V vs. RHE.

The Tafel analysis and Koutechky–Levich analysis were carried out in order to evaluate the kinetics of the ORR. For Tafel curves, $j_{k}$ can be obtained by normalizing the RDE area by $I_{k}$. $I_{k}$ is calculated through Formula (2). Then, the Tafel slope is calculated by the curve slope of E ~ log $\left|j_{k}\right|$.

The number of electrons transfer can be evaluated by K-L formula [47,48].

(3) $$\frac{1}{j}=\frac{1}{j_{k}}+\frac{1}{j_{d}}$$

where, j is the current density, $j_{k}$ is the kinetic current density and $j_{d}$ is the diffusion current density.

(4) $$j_{d}=0.2\mathrm{nF}C_{O_{2}}D_{O_{2}}^{2/3}\nu^{-1/6}\omega^{1/2}=B\omega^{1/2}$$

(5) $$B=0.2nFC_{O_{2}}D_{O_{2}}^{2/3}\nu^{-1/6}$$

where B is Levich constant, n is the number of electrons transferred per O_2_ molecule, F is the Faraday constant (96500 C/mol), $C_{O_{2}}$ is the concentration of dissolved oxygen in 0.1 M HClO_4_ (1.26 × 10^−6^ mol/cm^3^), $D_{O_{2}}$ is the diffusion coefficient of dissolved oxygen (1.93 × 10^−6^ cm^2^/s), ω is the RDE rotation speed (rpm) and ν is the kinematic viscosity of the electrolyte (1.009 × 10^−2^ cm^2^/s) [49]. Then, the *n* can be calculated by Formula (5).

### 3.5. Electrocatalyst Durability Characterization

The accelerated durability test (ADT) was performed by cyclic potential sweeps between 0.6 V and 1.1 V vs. RHE at sweep rate 100 mV/s for 5000 cycles in 0.1 M HClO_4_ electrolyte solution at room temperature. The same three-electrode system was used except a carbon stick was utilized as the counter electrodes. Then, the ECSA and ORR activities were obtained and compared with the performance of 20% Pt/C catalyst which after durability test.

The accelerated durability test for start-stop was performed by square wave potential cycles (accelerated durability test square wave cycling, ADT-SWC), stepped the potential between 0.4 and 1.5 V vs. RHE for 5000 cycles and held the potential at each value for 1 s [38,50]. The whole test was in 0.1 M HClO_4_ electrolyte solution at room temperature. The same three-electrode system was used except a carbon stick was utilized as the counter electrodes. Then, the ECSA and ORR activities were obtained and compared with the performance of 20% Pt/C catalyst which after ADT-SWC.

## 4. Conclusions

In this work, a hollow-structure Pt-Ni electrocatalyst is synthesized by sacrificial SiO_2_ template method. The structure of the electrocatalyst is a hollow sphere where the diameter is about 370 nm and the thickness is about 35 nm. It shows a higher ORR activity than that of the commercial Pt/C catalyst. At 0.9 V vs. RHE, the specific activity is 1.88 mA/cm^2^, which is higher than that of the Pt/C, the mass activity is 0.49 A/mg, which is about 7 times that of the Pt/C. The Tafel slope is 65.85 mV/dec, lower than that of the Pt/C. Moreover, it also shows that the electrocatalyst is controlled by a combination of two-electron and four-electron pathway through the K-L analysis. These results indicate that the hollow structure can improve the ORR activity of the catalyst effectively. Meanwhile, the XPS spectra shows that the addition of Ni results in a negative Pt binding energy shift of 0.56 eV, which promotes the oxygen reduction reaction activity of electrocatalyst.

More significantly, it has a higher durability than Pt/C against both ADT and the start–stop process. The SA and MA after ADT only dropped by 13.8% and 28.5%, respectively, indicating that the hollow structure can avoid movement, aggregation and Ostwald ripening during the ADT process. The SA and MA after ADT-SWC dropped by 34.6% and 40.8%, respectively, which are far better than those of commercial catalysts, suggesting that carbon-free electrocatalyst can avoid carbon corrosion during the start–stop process and slow the degradation of activity.

## Figures and Tables

**Table 1:** Current density at 0.9 V vs. RHE, $I_{k}$ , half-wave potential, MA and SA of Pt/C and hollow-structure Pt-Ni electrocatalyst before and after 5000 cycles of ADT and ADT-SWC.

Catalyst	Current Density at 0.9 V vs. RHE (mA/cm^2^)	*I_k_* (A)	Half-Wave Potential (V)	MA (A/mg)	SA (mA/cm^2^)
Pt/C	1.02	2.39 × 10^−^^4^	0.847	0.07	0.10
Pt/C (ADT)	0.01	1.86 × 10^−^^4^	0.719	0.04 (0.8 V vs. RHE)	0.08 (0.8 V vs. RHE)
Pt/C (ADT-SWC)	0.1	2.03 × 10^−^^5^	0.662	0.03 (0.8 V vs. RHE)	0.09 (0.8 V vs. RHE)
Hollow-structure Pt-Ni	2.89	1.94 × 10^−^^3^	0.906	0.49	1.88
Hollow-structure Pt-Ni (ADT)	2.75	1.39 × 10^−^^3^	0.914	0.35	1.62
Hollow-structure Pt-Ni (ADT-SWC)	2.62	1.15 × 10^−^^3^	0.891	0.29	1.23

## Data Availability

The data generated or analyzed during the study are included in the article.
